# Remembering and Communicating Climate Change Narratives – The Influence of World Views on Selective Recollection

**DOI:** 10.3389/fpsyg.2019.01026

**Published:** 2019-05-07

**Authors:** Gisela Böhm, Hans-Rüdiger Pfister, Andrew Salway, Kjersti Fløttum

**Affiliations:** ^1^Department of Psychosocial Science, Faculty of Psychology, University of Bergen, Bergen, Norway; ^2^Department of Psychology, Inland Norway University of Applied Sciences, Lillehammer, Norway; ^3^Institute of Experimental Industrial Psychology (LueneLab), Leuphana University of Lüneburg, Lüneburg, Germany; ^4^Sussex Humanities Lab, University of Sussex, Brighton, United Kingdom; ^5^Department of Foreign Languages, Faculty of Humanities, University of Bergen, Bergen, Norway

**Keywords:** climate change, world views, narratives, story telling, constructive memory, audience effects, computational text analysis

## Abstract

We examine how people remember stories about climate change and how they communicate these stories to others. Drawing on theories of reconstructive memory and cultural theory, we assume that recollection is systematically affected by an individual’s world view as well as by the world view of the target audience. In an experimental study with a Norwegian representative sample (*N* = 266), participants read a story about three politicians, in which each protagonist was described as holding a specific world view and as trying to tackle climate change with a corresponding strategy (individualistic/free market oriented, hierarchical/technology-oriented, or egalitarian/sustainability-oriented). After 1 day and then after 1 week, participants were asked to retell the story as if to somebody who was characterized as being either an individualist, a hierarchist, or an egalitarian; in addition, a neutral recall control condition without a specified audience was included. Participants’ own world view was assessed and they were classified as endorsing individualism, or hierarchism, or egalitarianism. We hypothesized that retellings would be selectively reconstructed according to the world view of the participant, as well as tuned to the audience’s world view. We assessed the cognitive structure of the recollected story, and, using methods from computational text analysis, we computed similarities among retellings and the original narrative, and among retellings and world views. Results suggest that (i) retellings become less accurate over time, (ii) retelling to an audience with an explicit world view leads to more strongly filtered retellings than recalling without a specified audience, but the filter operates in a non-specific manner with respect to world views, (iii) the cognitive structure of the recollected story shows small but systematic differences concerning the link between story problem and solution as a function of the participant’s and the audience’s world view. No interaction was found between the world view of the participant and that of the audience. Results emphasize the role of world views in communicating climate change, and might help to better understand phenomena such as polarization and echo chamber effects.

## Introduction

Since Bartlett’s seminal work on constructive memory (Bartlett, 1932), many studies have shown that people’s recall of narrative information is not a literal record, but tends to be reconstructed according to one’s acquired cultural knowledge. Recollections are not simply subject to random forgetting, but are the result of systematic modifications and alterations. The constructive aspect of memory has been explained by processes such as conventionalization, rationalization, simplification, assimilation, and distortion (Wagoner, 2017). What people typically remember is strongly influenced by the categories and schemata they utilize when interpreting their experiences.

Remembering also has a communicative function. We talk about our experiences, and we like to share our memories with others (Hirst and Echterhoff, 2012). Stories are the main carriers of socio-cultural knowledge and serve a social as well as an informative function (Schank and Abelson, 1995). In the process of telling stories to others, people take into account the kind of audience they are addressing; what people tell to others is influenced by their own intentions, for example, wanting to inform or to persuade, but is at the same time influenced by features of the audience they talk to, such as attitudes, expectations, or preferences (Marsh, 2007).

In this study, we apply the constructive memory framework to climate change narratives (Jones and Song, 2014; McBeth et al., 2014; Fløttum and Gjerstad, 2017; Sharman and Howarth, 2017). Concerning the climate change debate, a distinction often made is between people who endorse the assumption that climate change is happening and mainly of anthropogenic origin, and those who question that climate change is real or who consider it to be an entirely natural phenomenon (Poortinga et al., 2011; Hamilton et al., 2015). For the sake of brevity, we call these positions advocates and skeptics, respectively. Advocates and skeptics show substantial differences in their world views and political orientation, and in their beliefs about the causes and consequences of climate change. Between these camps, an increasing polarization has been observed, though the degree of polarization differs considerably between countries (Hoffman, 2011; Dunlap et al., 2016; Ceglarz et al., 2018).

Polarization may partly be attributable to so called ‘echo chambers’ or ‘filter bubbles’; these metaphors mainly refer to social media such as Facebook and Twitter, where like-minded individuals communicate with each other, reinforcing their respective stance and evaluation of climate change (Jasny et al., 2015; Williams et al., 2015). Retelling a story about climate change to people who essentially agree with one’s view may bolster this view in two ways: first, the speaker, holding world view X, adapts her retelling to her own world view; since the speaker and the listener hold the same world view, no conflict arises and the speaker’s statements are likely to be accepted and reinforced by the listener. Second, the speaker tunes her retelling to the listener’s world view X, and thus constructs a version that even more conforms to her own world view X. In a communication between like-minded, adaptation and tuning both enhance the correspondence between the communicators’ world views and the content of the communication. We propose that such processes of constructive memory may partly account for the ‘echo’ in echo chambers, and for the polarization of the climate change debate.

We report a study using methods from computational text analysis (Welbers et al., 2017) to analyze participants’ recollections of a narrative about climate change. This study contributes to a growing line of research which uses natural language texts or open-ended textual responses, that is, ‘text as data’ rather than quantitative survey questions, to investigate how people understand and evaluate climate change (Grimmer and Stewart, 2013; Paschen and Ison, 2014; Tvinnereim and Fløttum, 2015; Fløttum, 2017; Salway, 2017; Tvinnereim et al., 2017). Most research using text analysis employs a bottom-up approach, inducing regularities in massive collections of texts from online sources (Facebook, Twitter, etc.) by automated classification methods, such as clustering or topic modeling (Grimmer and Stewart, 2013; Roberts et al., 2014; Tvinnereim and Fløttum, 2015). We apply computational text analysis to data from an online experiment, analyzing quantitative characteristics of texts as a function of experimental manipulations in order to test hypotheses (Roberts et al., 2016).

Specifically, we test how the speaker’s view and that of the audience shape how a story with a climate change theme is recollected and retold. In addition, we test how these recollections change over time. We hypothesize that story retellings will be adapted to the speaker’s as well as to the listener’s world view, and that the conformity will increase over time.

## Theoretical Background

### Stories and Narratives

According to Schank and Abelson (1995), stories constitute the fundamental component of human knowledge. Stories are continuously heard from others, and told and retold to others, thereby constructing an individual’s representation of his or her self and of the world (Beach et al., 2016). Stories typically follow a schematic structure (Mandler and Johnson, 1977; Mandler and Goodman, 1982). The narrative policy framework (Jones and McBeth, 2010; McBeth et al., 2014) distinguishes a setting, a plot, characters, and a moral or a solution to a problem. The characters are categorized as villains causing the problem, as victims being harmed, and as heroes solving the problem. Related approaches can be found in text linguistics (Fløttum and Gjerstad, 2013, 2017; Beach et al., 2016). Stories typically refer to an individual’s experiences, whereas generalized stories that address common social or political phenomena are often called *narratives* (Mairal, 2008; Jovchelovitch, 2012; McBeth et al., 2014; Brown, 2017). In this paper, we do not strictly distinguish between stories and narratives, but use both terms for the most part synonymously.

### Climate Change Narratives and World Views

A climate change narrative represents people’s understanding of the climate change issue, including political and scientific aspects. Whereas the vast majority of scientists agree about the scientific evidence concerning climate change, its dynamics and its causes (IPCC, 2014), popular narratives may range from outright disbelief, regarding climate change as a hoax contrived by left-wing ecologists, to viewing climate change as the most pressing problem of humankind, caused by greedy and reckless capitalists. Evidence suggests that narratives rather than scientific facts represent people’s understanding of climate change (Lowe et al., 2006; Fløttum and Gjerstad, 2013, 2017; Jones, 2014; Paschen and Ison, 2014; Brown, 2017). Important elements of narratives are (a) causal relations (e.g., what are the causes of climate change, how can climate change be mitigated?), (b) intentions of relevant actors (e.g., who is responsible for climate change?) (Böhm and Pfister, 2001, 2005, 2008; Bostrom et al., 2012), and (c) affective and moral evaluations of strategies to mitigate climate change as well as of consequences of climate change (Böhm et al., 2018; Doran et al., 2018).

Studies by Guber (2013) and Jones (2014) suggest that people’s understanding of climate change is strongly based on their world views. World views can be considered as general cultural schemata which serve to assimilate particular experiences and stories about an issue such as climate change. We conceive of world views as serving the role of *cultural schemata* sensu Bartlett (1932), which control the way specific instances of stories are interpreted and adapted. Recent research has documented that world views as conceptualized in cultural theory (Douglas and Wildavsky, 1982; Thompson et al., 1990) play a significant role in climate change discourses and help to better understand the respective political debates about causes and strategies (Jones and Song, 2014).

We conceive of world views as the primary cultural schemata that shape how people understand social issues such as climate change. We employ the typology proposed by cultural theory (Douglas and Wildavsky, 1982; Thompson et al., 1990; Verweij et al., 2006) comprising four principal world views, each view representing a different stance concerning social relations and the relation between humans and nature: (i) The *egalitarian* view considers nature as fragile and unstable, and humans as being responsible for behaving in a sustainable way; this view is associated with a more liberal/left political orientation. (ii) The *hierarchical* view considers nature as basically stable, but vulnerable to human activities; a hierarchist relies on science and technical experts to solve problems and is moderately associated with a conservative political orientation. (iii) The *individualistic* view considers nature as robust and stable and largely immune to human activities, as long as nobody and no higher power interferes; this view is associated with a conservative political orientation, favoring free-markets and individual freedom. (iv) The *fatalist* view considers nature as well as society as unpredictable and humans as unable to influence the course of events; politically, a fatalist tends to be non-political and to refrain from political action, thinking that nothing can be done anyways. For variants on this typology see Kahan et al. (2011) or Kahan (2012).

Following Jones and Song (2014), we retain this classic approach in our study because it has proven to be a useful typology in climate change research (Jones, 2014; Jones and Song, 2014). We exclude the fatalist world view, for practical reasons and following an argument by Verweij et al. (2006; Jones and Song, 2014) that fatalists do not consistently participate in public debate about climate change simply because they are fatalists, and thus do not form a coherent schema that may influence recollections.

Typically, the type of narrative people endorse and people’s political orientations are correlated (McCright et al., 2016; Ziegler, 2017). Individuals with a left-leaning political orientation (socialists, democrats, liberals, etc.) tend to show stronger belief that climate change is happening and caused by humans, and to show stronger support for strategies to mitigate climate change, than individuals with a right-leaning political orientation (conservatives, republicans, neo-liberal free-market advocates, etc.). We will take up the role of political orientations in the discussion.

### Audience Effects

Conversation is not a simple process of transmitting information. It follows rules that take into account characteristics of the speaker and the listener, and the common knowledge of speaker and listener (Grice, 1975). Much if not most of remembering occurs during social interactions and conversations, and is thus shaped by both individual memory processes (Roediger and DeSoto, 2015) and conversational rules. Depending on the social situation, what is remembered is changed and adapted to the affordances of the situation, including features of the audience (Hirst and Echterhoff, 2012; Rechdan et al., 2016). Remembering in a social context can thus be understood as a *co-construction* process (Pasupathi, 2001), whereby speakers are influenced by their schemata as well as by the requirements of the context and the expectations of the audience (Grice, 1975). Co-construction is an adaptive process characterized as audience tuning by Hellmann et al. (2011).

Thus, retelling a story to an audience is quite different from recalling a memory in isolation (Marsh, 2007). Retelling a story repeatedly alters the content and structure of the story progressively, because what is retold strengthens the memory traces of the retold information, and information that was not retold decays (Anderson et al., 2000). Repeated retelling makes the retold narrative increasingly coherent and conforming with the reteller’s schema as well as with the audience schema; as a result, a retold story becomes simpler and its similarity to the original story or experience declines, while at the same time becoming increasingly coherent with respect to the endorsed cultural schemata. Echo chambers are a suitable metaphor describing this mutual reinforcing effect in the context of social media communities.

## Research Questions

Based on the theoretical background outlined above, we assume that climate change stories are shaped by the world views of people who tell such stories, as well as by the world views of the audience to whom the stories are told. World views are assumed to work as *reconstruction filters*, modifying stories when they are repeatedly told and retold. To our knowledge, the role of world views in memory and communicative processes in the context of climate change has not yet been studied. The investigation of audience world views and their interaction with speakers’ world views, in combination with applying computational text analysis, thus contributes to the novelty of this study.

Three research questions will be examined in more detail:

First, since reconstructive filtering implies simplification, we assume that over time stories will become simpler and thus less similar to the original story (*time effect*).
Second, a *schema conformity effect* is expected: stories will adapt to the speaker’s world view (*speaker effect*) as well as to the audience’s world view (*audience effect*). In particular, if both world views match, this effect will be especially strong and might possibly account for an echo chamber phenomenon.
Third, without an audience, retelling a story will basically be an isolated recall task; we assume that any filter mechanisms will apply to a significantly lesser degree in this situation compared to when an audience is present (*control group*).

To address these research questions, we included the speaker’s and the audience’s world view as independent variables. In addition, we had participants retell the story at two points in time. As there exists not the one accepted and valid method to assess story content and structure, we applied three approaches complementing each other: A sorting task of story related concepts (Coxon, 1999), and two methods from computational text analysis: similarity analysis (Kjell et al., 2019) and dictionary analysis (Welbers et al., 2017); for details see the method section.

## Materials and Methods

An online study was conducted with a representative Norwegian sample. All manipulations and measurements were conducted online.

### Participants

The data collection was conducted by a commercial research company (Norstat). Participants were recruited from their online panel of adult (18 years and older) Norwegian citizens. The final sample consists of 266 participants that had completed all three stages of the study (presentation of the story and retelling at two points in time). Their age ranged from 18 to 82 years (*M* = 44.5, *SD* = 15.8); 121 were female and 145 male; 176 held a Bachelor or higher university degree.

Participants were recruited online. At the start of the study, participants were informed about the topic and aims of the study, the anonymity of their answers, and the right to withdraw at any time from their participation. Participants gave their consent to take part by clicking a button when following the link to the questionnaire.

### Design and Procedure

The experiment consisted of three stages. At Stage 0, all participants read the same original story (OS) about three politicians who set out to solve the climate change problem, but with very different strategies, leading to different consequences (see Supplementary Material). Participants later recollected the OS at two points in time, at Stage 1 after 1 day, and at Stage 2 after 1 week. At both points in time, there were four retelling conditions. Participants were either asked to recall the story in as complete a manner as possible (control condition without audience), or they were asked to retell the story to an audience holding a specific world view, that is, to a person who was depicted as either a typical individualist, hierarchist, or egalitarian. At the end of Stage 2, a questionnaire was administered measuring the participant’s world view. Based on this questionnaire, participants were clustered into three distinct world view groups, representing individualism, hierarchism, or egalitarianism.

The experimental design is a 2 (Time) × 4 (Audience) × 3 (World View) three-factorial design, with a repeated measurement factor Time (two levels: Stage 1 and Stage 2), a between-subjects factor Audience (four levels: control, individualist, hierarchist, and egalitarian), and a quasi-experimental between-subjects factor World View (3 levels: individualism, hierarchism, and egalitarianism). Three types of dependent measures were taken: the recollected text served as the main dependent variable, a sorting task was used to measure the participants’ cognitive representation of the story, and a set of further rating scales were used to assess participants’ political orientation and attitude toward climate change.

### Materials

#### The Original Climate Change Story

The main stimulus material consisted of a text (604 words) about three politicians who set out to tackle the problem of climate change (see Supplementary Material). One character Tom Brown was portrayed as a conservative relying on a technical solution, closely corresponding to a hierarchist’s world view. A second character Matt Greene was portrayed as a left-wing politician with an egalitarian world view, trying to enforce stricter laws prohibiting unsustainable consumption. Bob Wayne, the third character, was portrayed as a free-market advocate with a typical individualist’s world view, who promoted establishing free trade so that market forces would eventually solve the climate change issue. All three politicians encountered serious problems trying to implement their strategies; when finally some success showed up, a dispute arose among the three characters about whose strategy it was that was effective. The story ended with all three politicians being assassinated (the reader may recognize a reminiscence of Bartlett’s classic story War of the Ghosts).

The story was intended to present a combination of three strategies to counter climate change, each strategy corresponding to a world view from cultural theory (individualism, hierarchism, and egalitarianism). This provided the opportunity for participants to select information corresponding to the audience’s and their own world view. Due to the length and complexity of the story, after only one reading a substantial amount of forgetting was to be expected, providing room for selective recollection processes.

#### Measures

At the end of Stage 2, the participant’s world view was assessed, which served to classify each participant as belonging to one of three world view groups. The recollection of the original story was assessed twice, at Stage 1 and at Stage 2. A sorting task was used to assess the cognitive representation of the story after Stage 2. Finally, at the end of the experiment, a set of judgments about political orientation and climate change attitude was elicited.

##### World views

We assessed world view using a 20-item questionnaire in Norwegian adapted from Grendstad (2001; see Supplementary Material). Each world view was measured by five items, yielding scales for individualism, hierarchism, egalitarianism, and fatalism. Based on all 20 items, participants were grouped via cluster analysis (hierarchical-agglomerative using Ward’s clustering algorithm) into three distinct clusters, corresponding roughly to the individualist, hierarchist, and egalitarian type. This one-to-one mapping of participant to world view serves as an approximation, in reality, most people do not represent pure types. Following Jones and Song (2014) we omitted the fatalism world view (see section “Climate Change Narratives and World Views”).

##### Sorting task

The sorting task aimed to provide a measurement of the cognitive structure of the story as mentally represented by the participant. The procedure closely followed that of Jones and Song (2014). Participants saw a list of 30 terms that were related to the original story. Participants were asked to carefully read the list of terms and then to sort them into boxes so that terms that belonged together in the story were placed together in the same box. The sorting task was done on screen via drag and drop. Participants were free to group all or only some terms into boxes, and to choose how many boxes to use. From the sorting task, we created for each participant a symmetric co-occurrence matrix with 30 rows and 30 columns, representing the 30 terms, and a 1 in each cell *i, j* if the terms i and j were placed together in the same box (otherwise 0). The aggregated co-occurrence matrix was used as an indicator of the relations among the key terms of the story as represented by the participants.

##### Retellings

Each participant generated two recollections, the first 1 day after reading the original story, and the second after 1 week. Participants were asked to write down their recollection via keyboard in a text box shown on the screen. In the control condition, participants were asked to “write down how you remember the story, as completely as possible. It does not matter if you are uncertain about details.” Thus, the control condition constitutes a free recall task. In the treatment conditions, participants read a description of ca. 100 words of a person named Jon. Depending on the condition, Jon was portrayed as an egalitarian, a hierarchist, or an individualist (for full instructions see Supplementary Material). After reading the description, participants were instructed to imagine what kind of person Jon is, and then asked to retell the story in a manner they would tell it if Jon were actually listening. The instruction and the full description of Jon were presented at both Stages 1 and 2. All three experimental conditions constitute a hypothetical social situation in which the participant as speaker (reteller) interacts with a particular type of listener (audience); control and treatment can be seen as contrasting a recalling with a retelling situation (Marsh, 2007).

##### Story transportation

As controls, we measured transportation, that is, how seriously the story was read and how much participants were emotionally engaged. This was measured immediately after the story was read. Participants rated two questions: (a) ‘I was mentally involved in the story while reading it,’ and (b) ‘The story affected me emotionally’; both on a seven-point rating scale (1 = not at all, 7 = very much).

##### Additional variables

Participants answered a set of additional items which will not be analyzed here, such as judgments about the protagonists’ strategies, ratings of participants’ political orientation, and questions about climate change (all variables are included in the data set and script available online).

## Results

We will first report on the classification of participants into world view clusters; this assignment of participants to world views will then be used as a quasi-experimental factor. Second, the sorting task will be analyzed in order to examine how participants’ and audience world views are reflected in the participants’ cognitive representations of the story. Third, we look at the textual retellings and examine via computational text analysis how the retellings’ content changes as a function of the experimental factors (similarity analysis), and to what extent the retold stories conform to the speakers’ and the audience’s world views (dictionary analysis).

### World View Classification of Participants

To confirm the appropriateness of the four world view scales, a psychometric analysis of the 20 world view items was performed, yielding a clear four-factorial structure, explaining 39% of the variance (maximum likelihood factor analysis with varimax rotation, RMSEA = 0.049). Cronbach’s alpha was 0.78 for individualism, 0.72 for egalitarianism, 0.62 for hierarchism, and 0.67 for fatalism.

Participants were then classified as individualist, hierarchist, or egalitarian, based on a cluster analysis. A distance matrix using Euclidean distances between all participants was computed from the matrix of *z*-scaled world view items. The distance matrix was subjected to a hierarchical-agglomerative clustering using Ward’s algorithm (Everitt et al., 2011). The three-cluster level was selected to assign participants uniquely to one cluster, yielding an almost equal distribution of participants across clusters (individualism: 86, hierarchism: 90, and egalitarianism: 90). The mean scale value for each world view was computed for each cluster, and a cluster was labeled according to the maximum value of the world view scales (Table 1).

**Table 1:** Means of world view scales for three clusters.

	Cluster		
	
	1	2	3
	IND	HIER	EGAL
Individualism	*5.02*	4.80	3.43
Egalitarianism	4.17	5.26	*5.72*
Hierarchism	4.21	*4.55*	3.64
Fatalism	3.11	3.86	2.80


*Maximum value for each world view is in italics.*

**FIGURE 1:**
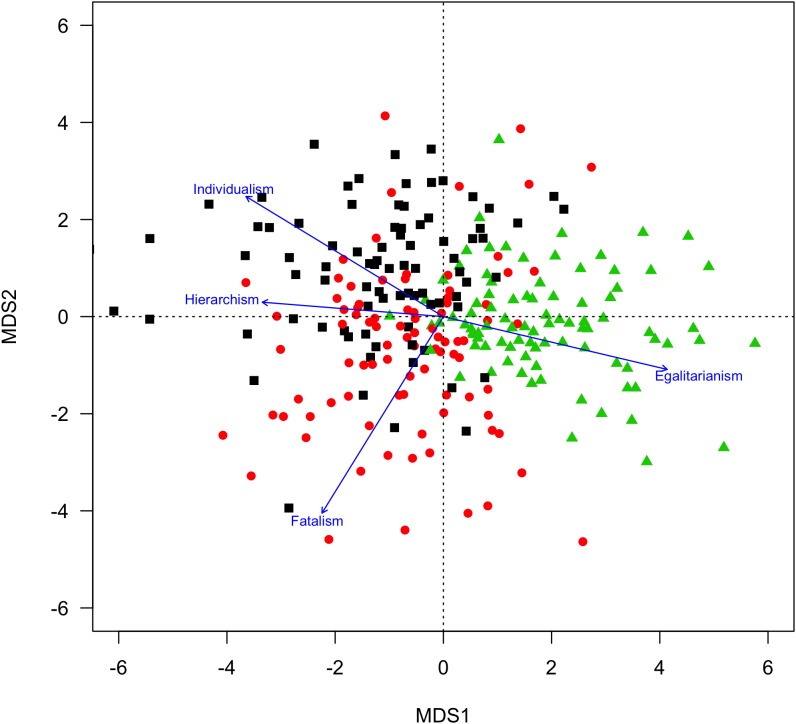
Multidimensional scaling analysis of participants, based on their distance on world view items. Participants as points (black squares = individualists, red circles = hierarchists, green triangles = egalitarians), and world view scales fitted as directional vectors.

Individuals in each cluster are in fact mixtures of all world views. Figure 1 depicts the similarities of the participants in a two-dimensional plane (fitted via ordinal multidimensional scaling of the distance matrix), with the world view scales fitted as directional vectors (Borg and Groenen, 2005). Each point represents a participant, and the projection of the point on a world view vector indicates how characteristic this world view is for this participant. The individualism and the hierarchism cluster appear as opposite to the egalitarianism cluster, and the hierarchism cluster shows substantial overlap with the individualists. Table 1 shows that Cluster 2 is the cluster with the highest score on the hierarchism scale, though this is still less than the scores on both the individualism and the egalitarianism scale for this cluster. Note that this cluster assignment serves the aim to construct a quasi-experimental factor discriminating participants according to the three world views as defined *a priori* for experimental purposes.

### Story Representation Measured by the Sorting Task

The individual co-occurrence matrices (see section “Sorting task”) for the sorting task terms were aggregated with respect to each condition of the Audience factor and of the World View factor. The aggregated matrices were converted to distance matrices by subtracting each cell frequency from the maximum value (number of participants), and subjected to a hierarchical cluster analysis using Ward’s method (Everitt et al., 2011).

For interpretation, we inspected the dendrogram across all levels, closely following the approach applied by Jones and Song (2014). We interpreted aggregated clusters across Audience conditions and World View conditions, respectively, with respect to coherent sets of terms, and with respect to the role the terms play in the original story.

**FIGURE 2:**
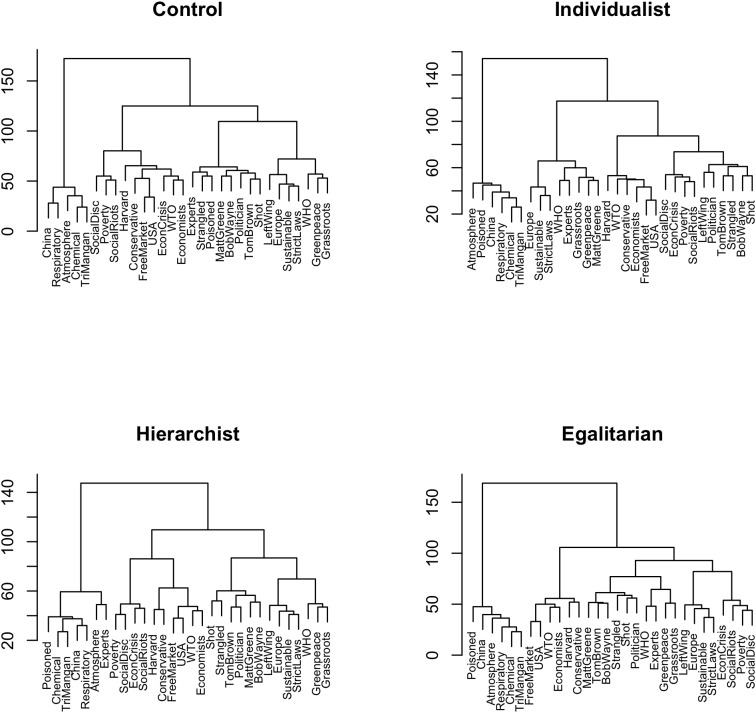
Cluster analyses of the sorting task for Audience conditions (Ward’s agglomerative algorithm based on co-occurrence matrices).

Figure 2 shows the cluster dendrograms for the Audience conditions. The control condition (pure recall) yields four discernable clusters (from left to right): A cluster with terms referring to the ‘chemical solution’ (CHEM for short), a cluster containing terms about the ‘free market’ solution (FM for short) and terms signifying ‘social crisis’ (CRIS for short), a cluster containing all protagonists of the story including their pitiful deaths (PROT for short), and a cluster representing the ‘sustainable solution’ (SUST for short). In an abbreviated form, we can write for the control condition

Control=CHEM+{CRIS+FM}+PROT+SUST.

The individualistic audience condition yields five clusters which are largely parallel to the control condition, but with an important difference: the free market cluster is less closely connected to the crisis cluster, which appears related to the protagonists; also note that Matt Greene is part of the sustainable solution cluster. In short

Individualist=CHEM+SUST+FM+{CRIS+PROT}.

The hierarchical audience condition is very similar to the control condition, again linking the crisis and the free market cluster, and can be written as

Hierarchist=CHEM+{CRIS+FM}+PROT+SUST.

The egalitarian audience condition shows an analogous structure, but with notable differences. The crisis cluster is closely connected to the sustainability cluster, and the terms referring to the sustainable solution are grouped into a subcluster representing institutions (WHO, etc.), and another subcluster representing the political strategy (strict laws, etc.). In short

Egalitarian=CHEM+FM+PROT+[{SUST_inst+SUST_strat}+CRIS].

The audience effect shows mainly in the location of the crisis terms. In the recall condition, crisis is associated with terms indicating a free market solution; a similar clustering occurs for the hierarchist condition. Retelling the story to an individualist locates the crisis together with the story’s protagonists, and retelling to an egalitarian locates the crisis close to the sustainable solution.

**FIGURE 3:**
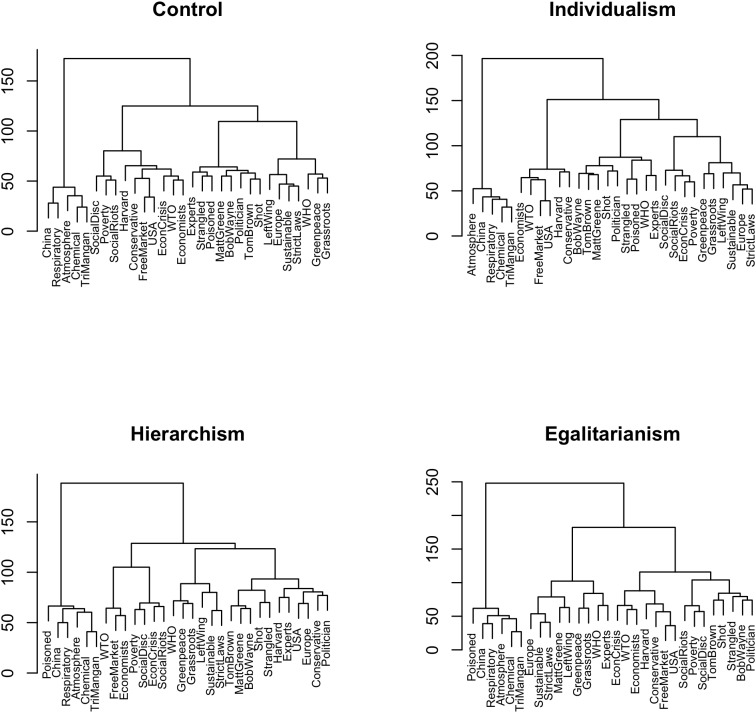
Cluster analyses of the sorting task for World View conditions (Ward’s agglomerative algorithm based on co-occurrence matrices). Note that the upper left dendrogram is from the Audience control condition, repeated from Figure 2 to facilitate comparison.

Comparing cluster dendrograms across World View conditions reveals a complementary pattern of crisis-solution associations (Figure 3). We find for individualism a structure with a close connection of crisis and the sustainable solution. The protagonist cluster interestingly contains the WHO and the experts, subsuming abstract institutions as protagonists. In short

Individualism=CHEM+FM+PROT+{CRIS+SUST}.

Participants with a hierarchical world view show an interesting deviation. In addition to the typical clusters shown in the other conditions, a new cluster emerges containing terms such as ‘expert,’ ‘politician,’ and ‘Harvard.’ We label this cluster the expert cluster (EXP), possibly reflecting the hierarchist’s view of the world as hierarchically structured with some kind of experts as a special group of people. Also, the crisis cluster is closely connected to the free market solution. In short

Hierarchism=CHEM+{FM+CRIS}+SUST+PROT+EXP.

Egalitarians yield a close connection of crisis, free market, and the protagonists of the story (except the egalitarian protagonist Matt Greene). As was found for the audience egalitarian condition, the sustainable cluster is divided into two subclusters, one containing institutions such as Greenpeace, one containing terms indicating strategies such as stricter laws. In short

Egalitarianism=CHEM+{SUST_strat+SUST_inst}+{FM+CRIS+PROT}.

As in the Audience conditions, the World View conditions differ mainly in the location of the crisis cluster. Individualistic participants see crisis as associated with sustainable solutions, hierarchical participants with free markets, and egalitarian participants with free markets and protagonists.

### Computational Text Analysis of Retellings

We obtained 532 = 2 × 266 recollections in textual form. For the original Norwegian texts, the mean number of words was 78.9 (*SD* = 68.4, *Median* = 59), with little difference between Stage 1 (*M* = 80.1) and Stage 2 (*M* = 77.7). Six recollections had zero words. For all analyses, the Norwegian texts were automatically translated to English, using the RYandexTranslate package (Chaware, 2016) for the R Computing System (R Core Team, 2018). For the translated English texts, the overall mean number of words was 85.3 (*SD* = 75.2, *Median* = 64); for Stage 1 texts, the mean was 86.7, and for Stage 2 the mean was 83.9.

The story transportation measures indicated that participants felt moderately to highly involved while readings the story, *M* = 4.72 (*SD* = 1.50, *Median* = 5, on the 7-point scale), and that they were moderately emotionally affected by the story *M* = 3.68 (*SD* = 1.55, *Median* = 4).

The 532 translated text units served as the main text corpus. For analyses, texts were further processed using standard procedures such as lowercase conversion, deletion of stopwords, punctuation, and numbers, and stemming (Grimmer and Stewart, 2013; Welbers et al., 2017). According to the *bag-of-words* assumption, a text is viewed as a collection of words regardless of sequence and linguistic structures (Lucas et al., 2015); although this omits information, pertinent research has shown that this approach is able to capture much of the meaningful content. This reduction can be understood as a kind of normalization of texts, condensing natural text to its basic lexical content. For the reduced texts, mean word number was *M* = 40.9 per recollection (*SD* = 36.8, *Median* = 31), with little difference between Stage 1 (*M* = 41.5) and Stage 2 (*M* = 40.3). After normalization, 12 text units had zero words and were excluded from the following analyses.

#### Similarity Analysis

We expected that recollections become less similar to the original story over time, due to time *per se* (Hypothesis 1). Audience effects are expected to lead to higher similarity between the retelling and the original story in the control condition than when an audience exists (Hypothesis 3); if audience and speaker world views conform, reconstructive filtering is expected to be maximal, leading to particularly low similarity of the retelling to the original story (Hypothesis 2).

As a measure of document similarity, we used the cosine similarity between two texts (Thada and Jaglan, 2013; Günther et al., 2015). The collection of terms from all text units constitutes the vocabulary of the text corpus. A single text can be represented as a vector of frequencies across the vocabulary, that is, for each term in the vocabulary, the vector indicates how often that term shows up in the text. The matrix of all text vectors, with the texts as rows and the terms as columns, represents the document-term matrix (DTM), which is used as the basic data structure. Given two documents with text vectors *d*_1_ and *d*_2_ (two rows from the DTM), cosine similarity is defined as

$$s(d_{1},d_{2})=\frac{d_{1}\cdot d_{2}}{\left|d_{1}|\times |d_{2}\right|}$$

Similarity ranges between 0 (no similarity, i.e., no common terms) and 1 (maximum similarity, i.e., identical text vectors). Cosine similarity *s*(*d*_i_, OS) was computed for all 532 recollections *d*_i_ with respect to the original story OS. Mean similarity was *M* = 0.15 (*SD* = 0.12; *Min* = 0, *Max* = 0.55).

**FIGURE 4:**
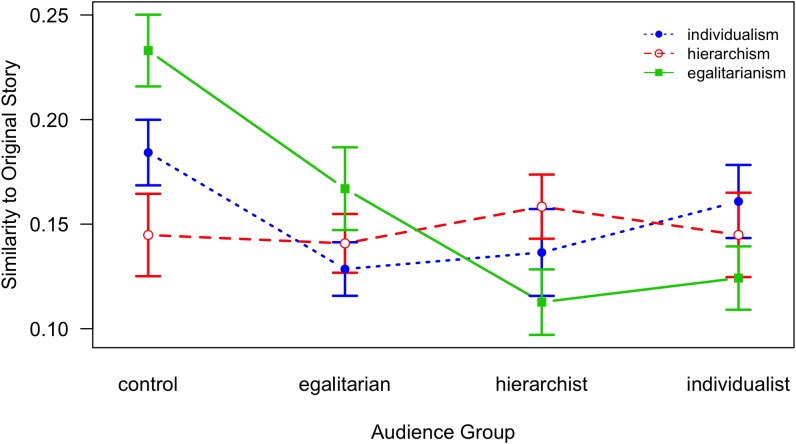
Similarity of a retelling to the original story as a function of Audience and World View; lines denote World View conditions (error bars indicate standard errors; note that the *Y*-axis is shifted).

**FIGURE 5:**
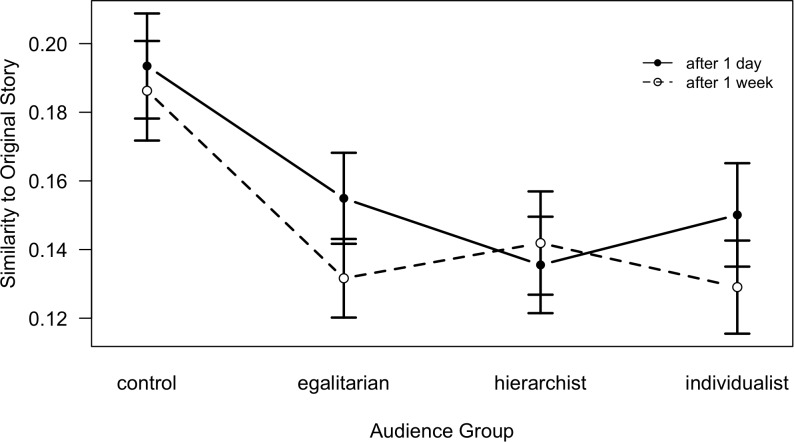
Similarity of a retelling to the original story as a function of Time and Audience; lines denote Time conditions (error bars indicate standard errors; note that the *Y*-axis is shifted).

An analysis of variance with similarity to the original story as the dependent variable and Audience (4 levels: Control, Egalitarian, Hierarchist, Individualist), World View (3 levels: Individualism, Hierarchism, and Egalitarianism), and Time (2 levels: Stage 1, Stage 2) as independent variables was conducted; Audience was varied between-subjects, Time was varied within-subjects, and World View was measured and served as a quasi-experimental between-subjects factor^1^. We found a main effect of Audience, *F*(3,254) = 3.06, *p* = 0.029, $\eta_{p}^{2}$ = 0.03, a main effect of Time, *F*(1,254) = 9.76, *p* = 0.002, $\eta_{p}^{2}$ = 0.04, and an interaction effect between Audience and Time, *F*(3,254) = 3.22, *p* = 0.023, $\eta_{p}^{2}$ = 0.04; overall *R*^2^ = 0.079. No other significant effects or interactions emerged. Similarity as a function of Audience and World View is depicted in Figure 4, similarity as a function of Audience and Time is depicted in Figure 5. With respect to the main effect of Audience (Figure 4, 5), contrast tests for the Audience factor showed that the control group was the only condition that differed significantly from the grand mean, *t*(254) = 2.99, *p* = 0.003, and, by implication, the control group differed from the three retelling conditions.

Hence, as can be seen in Figure 4, 5, the main effect of Audience is based on the difference between the control condition (pure recall) and the retelling conditions. In the control condition, similarity to the OS is generally greater than in any of the retelling conditions, except among participants assigned to the hierarchism world view (Figure 4); no differences emerged between the three different retelling conditions. In addition, though the pattern is not significant (*p* = 0.08), we can see from Figure 4 that for any world view, similarity is greatest if the person with that world view retells his or her story to a listener with the same world view, which is opposite to Hypothesis 2.

As expected, a significant effect of Time confirms that similarity to the original story generally declines over time (a 1 week interval). A simple effects analysis of Time across the levels of the Audience factor yields significant declines for the egalitarian condition, *t*(65) = 2.52, *p* = 0.014, and the individualist condition, *t* = 2.73, *p* = 0.008. The interaction effect between Audience and Time manifests as a significantly lower similarity at Stage 1 for the hierarchist condition, *t* = -2.58, *p* = 0.011 (Figure 5), in contrast to the other conditions.

In sum, similarity of retellings to the original story generally decreases over time and is higher if no particular audience is addressed than if an audience is imagined; however, no interaction of Time with the participants’ world view was found, *F*(2,254) = 0.72, *ns*. On the level of overall story similarity, the assumption that retellings are specifically tailored to the combination of speaker’s world view and the audience world view cannot be confirmed.

To obtain a more detailed assessment of story modifications that are specific to world views, we disentangled the original story into three story lines, one line for each central character. By construction, Tom Brown was portrayed for the most part as an individual with a hierarchical world view, Matt Green was portrayed as an egalitarian, and Bob Wayne as an individualist. Accordingly, for each character, only those text segments were selected that explicitly dealt with the actions of the respective character, yielding three partial stories (a Brown/hierarchist, a Greene/egalitarian, and a Wayne/individualist story). For each of the participants’ retellings, similarity to each partial story was computed, using the cosine similarity measure as before; these three similarity scores constituted a new repeated measurement factor Story Character, with three levels; for simplicity, we call the levels Brown (the hierarchist), Greene (the egalitarian), and Wayne (the individualist). According to our schema model we predict an interaction between Story Character and World View, as well as between Story Character and Audience. Similarity of a retelling to each of the story characters should be particularly large if the character’s world view corresponds to the participant’s world view, or to the world view of the audience.

**FIGURE 6:**
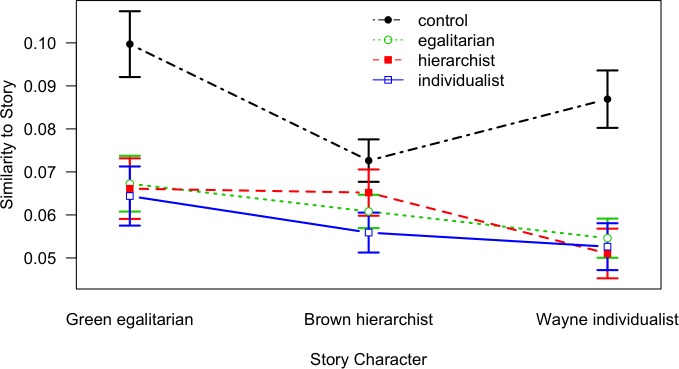
Similarity of a retelling to each of the story characters as a function of Story Character and Audience; lines denote Audience conditions (error bars indicate standard errors; note that the *Y*-axis is shifted).

**FIGURE 7:**
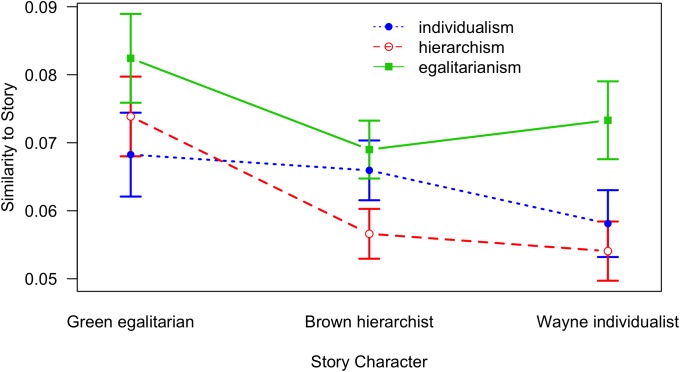
Similarity of a retelling to each of the story characters as a function of Story Character and World View; lines denote World View conditions (error bars indicate standard errors; note that the *Y*-axis is shifted).

An analysis of variance with similarity as dependent variable and Time and Story Character as within-subject factors, Audience as between-subjects factor and World View as a quasi-experimental between-subjects factor was performed (for technical details see Footnote 1). Results show significant main effects for Time, *F*(1,1305) = 4.95, *p* = 0.026, $\eta_{p}^{2}$ = 0.02, for Audience, *F*(3,254) = 3.34, *p* = 0.019, $\eta_{p}^{2}$ = 0.02, and for Story Character, *F*(2,1305) = 13.93, *p* < 0.001, $\eta_{p}^{2}$ = 0.04. Also, as predicted, significant interactions were obtained between Story Character and Audience, *F*(6,1305) = 3.68, *p* = 0.001, $\eta_{p}^{2}$ = 0.03, and between Story Character and World View, *F*(4,1305) = 2.39, *p* = 0.049, $\eta_{p}^{2}$ = 0.01. Overall *R*^2^ = 0.085 (Figure 6, 7).

Figure 6 shows that it is mainly the control condition yielding largest similarities to all of the three story characters; the three retelling conditions (egalitarian, hierarchist, and individualist) are basically indistinguishable and yield lower similarities; this finding corresponds to the results from the previous similarity analysis and further suggests an unspecific filter mechanism. Also, the interaction between Story Character and World View is not as expected (Figure 7) according to our schema conformity hypothesis.

#### Dictionary Analysis

Another approach to check whether a text is related to a specific topic or theme is dictionary analysis (Welbers et al., 2017). Unlike similarity analysis, where similarity between two texts is defined across the entire vocabulary, dictionary analysis relies on a set of predefined words, and counts how often these words occur in a given text. If the collection of predefined words represents the essential meaning of a topic, a word count may serve as an indicator of how closely related the text is to the topic.

Accordingly, we classified the 30 terms from the sorting task (see section “Story Representation Measured by the Sorting Task”) in four categories, corresponding to the three world views (and, consequently, to the central story characters), and to a ‘crisis’ category (Table 2). A dictionary analysis then counts, for each retelling, how many words from each category are used. We can then specifically test for interactions of each category count with Audience condition and with World View condition.

**Table 2:** Word classification of the sorting task terms to four term categories.

Term category			

Individualist	Hierarchist	Egalitarian	Crisis
(Wayne)	(Brown)	(Greene)	
Wayne	Brown	Greene	Crisis
WTO	Strangle	Law	Riot
Harvard	Poison	Sustainable	Poverty
Conservative	Chemical	WHO	Social
Economy	Respiration	Left	Discrimination
Free	Mangan	Europe	
United States	Atmosphere	Grassroot	
Market	China	Strict	


We conducted a dictionary analysis using the four categories of words as shown in Table 2. Term Category was defined as a factor with four levels (individualistic, hierarchical, egalitarian, and crisis), each level referring to the words of that category. A word count yielded the frequency of terms from a Term Category included in a retelling. For counting, word stems were used (e. g., law^∗^ included all instances such as law, laws, lawful, etc.; computational details can be found in the analysis script available online). Each category consisted of eight terms, except the crisis category which comprised only five terms. To compensate, the word count for each category was inversely weighted by the number of category terms; these weighted counts entered in the following analyses.

An analysis of variance was performed with proportion of words (=weighted word count for a category, divided by the total number of words of a text) as dependent variable and Term Category, Time, Audience, and World View as independent variables, yielding significant main effects for Time, *F*(1,1786) = 6.17, *p* = 0.0129, $\eta_{p}^{2}$ = 0.04, Term Category, *F*(3,1782) = 92.85, *p* < 0.001, $\eta_{p}^{2}$ = 0.17, and Audience, *F*(3,249) = 3.073, *p* = 0.028, $\eta_{p}^{2}$ = 0.03, and a significant interaction between Term Category and Audience, *F*(9,1782) = 2.33, *p* = 0.013, $\eta_{p}^{2}$ = 0.02; overall *R*^2^ = 0.14 for the model.

**FIGURE 8:**
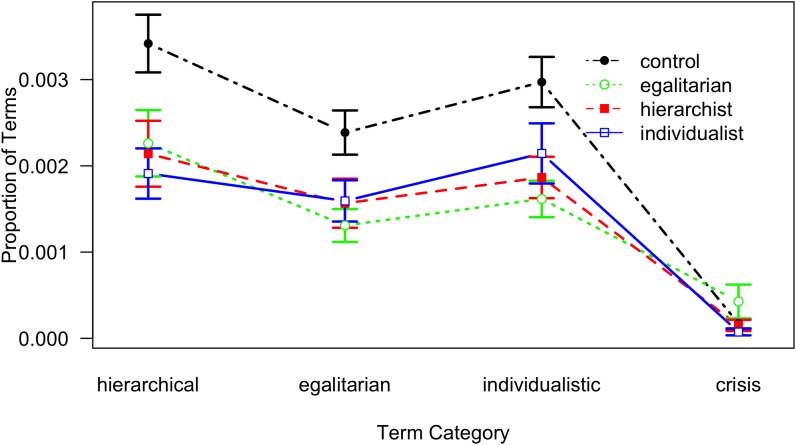
Proportion of words from each category as a function of Term Category and Audience; lines denote Audience conditions (error bars indicate standard errors).

As can be seen in Figure 8, the main effect of Term Category is manifested in a slightly higher proportion of words for hierarchical words, *t*(1782) = 8.63, *p* < 0.001, and individualistic words, *t*(1782) = 5.66, *p* < 0.001, and a substantially lower proportion of words from the crisis category, *t*(1782) = -15.16, *p* < 0.001. For Audience, only the control condition differs significantly from the overall mean, *t*(249) = 2.98, *p* = 0.003; however, these findings are qualified by the significant interaction, indicating that all differences between audiences disappear for the Crisis condition.

## Discussion

People’s evaluation of contentious social issues such as climate change is often only to a small degree influenced by their factual knowledge about these issues; cultural values and world views often play a stronger role in shaping such evaluations (Kahan et al., 2011). When people communicate about an issue, they tell and retell narratives that embody their beliefs as well as appraisals of the causes and evaluations of the consequences, conferring meaning to the issue (McAdams and McLean, 2013; Brown, 2017). One reason why these narratives and associated evaluations are so persistent and resist change might be due to memory processes which are at work during the communication process. Repeated telling and retelling of narratives can be expected to strengthen those aspects that are told and to weaken those aspects left out. Consequently, narratives may become more and more coherent and compatible with the person’s own core values and beliefs. Especially when conversing with like-minded people, a plausible assumption is that world views mutually reinforce each other and narratives are increasingly adjusted to these world views.

Climate change may serve as a prime example of this. Narratives about climate change provide explanations: whether the phenomenon exists at all, what its causes and its consequences are, what should be done. Narratives appear to be largely immune to scientific facts; given the large scientific consensus on climate change one might otherwise expect that all people would tell the same story. What happens is quite the contrary, narratives of climate change are quite diverse; some tell about villains who destroy the earth, others about conspiracies initiated in order to subdue the free world, and people align the moral of the story with their own basic beliefs and world views (Jones, 2014). Social media in particular may play the role of echo chambers, where communities of like-minded individuals mutually confirm their views about climate change (Jasny et al., 2015; Flaxman et al., 2016).

In this study we examined processes of constructive memory (Bartlett, 1932; Wagoner, 2017) as one possible factor shaping peoples’ climate change narratives. World views, we assume, serve as cultural schemata that operate as filters when people recollect narrative information and share their narratives with others. World views filter meaningful components of a narrative, sifting out what is not compatible with one’s world view. World views also operate when telling stories to others, in the sense that stories are tuned to the world view of the audience.

The focus of this study was on examining actual retellings, that is, texts generated by participants when asked to remember and retell a previously read narrative. We analyzed these natural language data by means of computational text analysis; specifically, we computed similarities between the retellings and the original story, between the retellings and partial aspects (story lines) of the story, and we computed the amount of specific world view-related topics occurring in the retellings via dictionary analysis (Welbers et al., 2017). Since what has been called the narrative turn in the social sciences, narratives have been mostly analyzed by qualitative methods (Riessman, 1993). In contrast, we attempted to quantify the main components of retold stories, and their interrelationships, aiming to capture important aspects of reconstructive processes. Additionally, we assessed the ensuing cognitive representation of the narrative by means of a more traditional sorting task method. Participants sorted the main terms from the narrative into groups, and a cluster analysis was employed to detect the underlying story representation from the derived co-occurrence matrices of terms.

The present study shows that world views exert a small though non-negligible influence on how climate change narratives are remembered and retold. An examination of the mental representation of a climate change story via a sorting task/cluster analysis approach revealed that although the general story structure is very similar across world views, the link between the problem component (a crisis due to climate change) and the proposed problem solutions (strategies to counteract climate change) varies systematically as a function of the audience’s world view and of the speaker’s world view. The audience effect indicates that speakers tune the retelling to the audience’s world view and connect the problem with that solution which is preferred by the audience; for example, retelling the story to an egalitarian who is assumed to prefer a sustainable strategy yields a close association between crisis and sustainable strategy. Also, the egalitarian structure contrasts to both the individualist and the hierarchist structure, the latter two being both more closely associated with a non-sustainable solution.

The effect of the speaker’s world view, in contrast, indicates that speakers tend to connect the aspects of the crisis with that strategy which – from their point of view – is the cause and culprit of the crisis. Thus, unlike the audience effect, which links the crisis with a solution, the effect of the participant’s world view is to link the crisis with the problem. Participants with an egalitarian world view closely associate the crisis with the free market, and with the respective protagonists. An individualistic participant, in contrast, associates the crisis with sustainable strategies. In sum, the findings from the cluster analyses suggest that the reteller’s world view links the story problem with the ‘villain’ (as seen from the reteller’s world view), whereas the audience effect tends to make the retelling compatible with the solution as seen from the audience’s world view.

The computational text analysis examined three hypotheses: first, a time effect was expected, that is, retellings were assumed to become less similar to the original story over time. Second, we expected a schema conformity effect, consisting of an audience effect and a speaker effect: Retellings were expected to become more similar to the speaker’s as well as to the audience’s world view, and consequently become less similar to the original story. Third, we expected that in the control group, retellings would be least affected by reconstruction processes, due to the absence of an audience.

The similarity analyses yielded a time effect, but only in the somewhat trivial sense that people forget when time passes. We found an audience effect, but that was unspecific: in the control condition, when participants simply recalled the original story without telling it to an audience, retellings were most similar to the original story. If an audience was present, retellings decreased in their similarity to the original story, but more or less to the same degree across the various audiences, irrespective of the specific world view held by the listener. In fact, the decay over time was not significant for the control/recall condition. We assume that world views of an audience do in fact operate as filters, but in an unspecific manner, introducing alterations and omissions unrelated to the audience’s specific world view, and distorting the original information in a somewhat random way. This finding suggests that talking to an audience of any kind activates a filter process so that the recollected story bears less resemblance to the original story than under pure recall without an audience. This is counter to our schema conformity hypothesis, which states that if speaker and listener share the same world view the filter effect would be amplified.

We find, however, some suggestive indication of schema conformity, though contrary to our hypothesis. Although the interaction between Audience and World View is not significant, *F*(6,254) = 1.9, *p* = 0.08, the interaction pattern suggests that identical world views of speaker and listener may increase story veridicality. When speaker’s and listener’s world views match, less filtering occurs and the narrative is more, rather than less, similar to the original story than when the world views of speaker and listener differ (Figure 4). A tentative explanation might be a common ground hypothesis (Clark and Brennan, 1991; Keysar et al., 1998), assuming that matching world views provide sufficient common ground for mutual understanding, making constructive processes and audience tuning superfluous. The motive to modify and adapt the recollection is low because no explanation or persuasion is needed and common ground is implicitly presumed. Interestingly, there is virtually no decline in similarity over time for the control/recall condition, suggesting that retelling to an audience not only decreases similarity due to audience tuning, but also makes the memory trace more fragile and amenable to deterioration over time.

Results of splitting up the story into its three story lines related to the three protagonists (Greene the egalitarian, Brown the hierarchist, and Wayne the individualist) are in line with the other findings. The control group yields largest similarities to all story characters, whereas the specific audience world views show no effect. However, an interesting interaction between story character and speakers’ world view emerges: those participants who hold an egalitarian world view produce the most similar recollections with respect to all three central characters of the story (Figure 7). Correspondingly, all world view conditions generate retellings that are most similar to the egalitarian character Matt Greene. This pattern might be due to the egalitarian world view being closest to the dominant politically correct view, as presumably seen by many people in Norway. A possible explanation could be that dominant world views operate as weak filters, generating little reconstructive modifications; conversely, minority world views might operate as strong filters and generate more substantial modifications and distortions.

The dictionary analysis largely confirms the findings from the similarity analysis. The pattern of word counts for different term categories does not support the hypothesis that those terms are recollected relatively more often which conform to the participant’s world view or to the audience’s world view. Instead, we find the opposite – no interaction between Term Category and World View, and the interaction between Audience and Term Category yields greater proportions for the control condition with respect to all categories, which is again in line with assuming an unspecific filter mechanism. In addition, the dictionary analysis shows virtually identical and very low proportions for all Audience conditions with respect to the crisis category. A plausible explanation might be that the crisis topic is a story feature that does not discriminate between the story characters and their strategies.

It should be noted that retelling a previously heard story is essentially a memory task and might be expected to substantially depend on age. However, including age as a covariate had virtually no effect on the results; in fact, age *per se* turned out to be not significantly related to any dependent measure. The task of retelling a semantically rich narrative is quite different from rote learning; although older people usually experience a decline in short term memory, memory loss is less pronounced or absent for personally relevant episodes and similar tasks (Schacter, 1996). Furthermore, since the amount of correctly remembered information from the original story was quite low generally, it could also be a floor effect obliterating age differences.

In sum, we find mixed evidence on how world views operate on the comprehension, recollection, and communication of climate change narratives. Results from the sorting task suggest that there is a small but systematic effect of world views on how people connect the problem of a story with its possible solutions. Results from the text analyses suggest that world views largely operate in a non-specific manner; irrespective of the audience’s world view, people modify their recollections whenever an audience is present (retelling conditions) in contrast to recalling a story in isolation (Marsh, 2007).

Several limitations of this study should be noted. The validity and reliability of narrative analyses has often been questioned (Riessman, 1993; Grimmer and Stewart, 2013; Brown, 2017). A direct written recording of recollections, as used in our study, yields extremely noisy data. After normalization, the mean number of words of a text unit was about 40, whereas the original story had approximately 600 words; some retellings consisted of just a few words. This constitutes an enormous loss of information that cannot be explained by a systematic influence of schemata on the reconstruction. The similarities between retellings and the original story are generally very low and the signal/noise ratio might be too low to detect substantial effects; especially when splitting the retellings according to the three story lines, this effect might be exacerbated. It may be questioned whether a completely open response format is capable of capturing the conceptual content of peoples’ recollection with sufficiently high precision; it may be that the requirement to articulate and write down one’s recollection simply generates a large amount of noise. Also, measuring similarity via a cosine similarity measure based on common word frequencies has its drawbacks. For example, in some conditions participants might rephrase the original wording using synonyms, which would lead to low similarity just because different words are used for the same concepts.

The sorting task, on the other hand, provides a kind of scaffold that guides recollection and blocks noisy intrusions, yielding a more stable measurement of the story’s representation, albeit in a less direct way. Providing the relevant concepts in the sorting task might artificially increase recollection, independent of experimental condition, and thus yield homogenous representations and obscure real differences. Also, it is somewhat unclear how strongly the sorting process depends on the recollected story; it might mainly be driven by the general world knowledge and understanding of the presented concepts. How to best measure narrative content and the dynamics of narrative change remains an open question and a challenge for future research.

Can memory processes explain echo chamber effects? If we view echo chambers as closed systems, that is, as a collection of like-minded individuals communicating only with each other, we would predict that any story would be distorted and fragmented over time, with little information remaining the same over time. However, the assumption that echo chambers operate as filter bubbles (Flaxman et al., 2016), systematically extracting compatible and distorting incompatible information, might be too strong. Our data indicate that the simple process of retelling, no matter to whom, generates a loss of information that is unsystematic. Furthermore, what we tentatively called a common ground hypothesis suggests that like-minded individuals have no motive to systematically reconstruct the stories that they tell each other, simply because they are like-minded and share a common world view in the first place. In contrast, when confronted with individuals from a different echo chamber, some adjustments in communicated content might well occur; our findings suggest that at least the key components of a story – Who is the villain? Who caused the problem? – might be constructed according to the speaker’s and the listener’s world views. These effects were small, though, in our study and they have also typically been small in other research (Jones and Song, 2014; Williams et al., 2015; Flaxman et al., 2016; Quattrociocchi et al., 2016). The existence of echo chambers appears to be an obvious phenomenon, and there is evidence that people tend to engage in social media activities with like-minded others, albeit not exclusively so (Williams et al., 2015; Flaxman et al., 2016). However, tapping into the specific processes that may lead to increasing polarization between echo chambers through transformation of discourse content, and studying these processes in a systematic scientific manner, may be more challenging than anticipated.

Potential implications of this study might go in two directions. First, we think that it contributes to the understanding of echo chamber effects, shedding some light on the role of reconstructive memory processes. Second, we hope to advance the understanding of computational text analyses in the study of narratives and to contribute to its methodological development.

## Ethics Statement

This empirical study complied with the Norwegian Social Science Data Services (NSD) privacy regulations and the ethical principles of research by the National Committee for Research Ethics in the Social Sciences and the Humanities (NESH). The NSD has approved that the project complies to these rules and regulations (project number 44066).

## Author Contributions

GB and H-RP contributed conception and design of the study. GB, H-RP, and AS performed the statistical analyses. H-RP wrote the first draft of the manuscript. H-RP and GB wrote sections of the manuscript. All authors contributed ideas to manuscript revision, read and discussed the manuscript, and approved the submission.

## Conflict of Interest Statement

The authors declare that the research was conducted in the absence of any commercial or financial relationships that could be construed as a potential conflict of interest.

## Footnotes

1 The model was estimated as a mixed-effects regression model with subjects as random factor (Judd et al., 2017), with the R package lme4 (Bates et al., 2015; R Core Team, 2018) using Satterthwaite’s approximation.
