# Supplementary material for: Remembering and Communicating Climate Change Narratives – The Influence of World Views on Selective Recollection
Source: Front Psychol. 2019 May 7;10:1026. doi: 10.3389/fpsyg.2019.01026 (PMC6514186; doi:10.3389/fpsyg.2019.01026)
Supplement: Supplementary file 1 [file Table_1.DOCX]

Supplementary Material

Remembering and communicating climate change narratives – the influence of worldviews on selective recollection

Gisela Böhm*, Hans-Rüdiger Pfister, Andrew Salway, Kjersti Fløttum

*** Correspondence:** Gisela Böhm: [gisela.boehm@uib.no](mailto:gisela.boehm@uib.no)

# The Original Story

## Original Norwegian Version

I 2025 bestemte en trio av ambisiøse politikere seg for å ta skikkelig tak i klimaproblemet. Ødeleggende stormer og tørke hadde økt på verdensbasis, noe som hadde ført til sultkatastrofer og økonomisk krise i store deler av verden. De tre politikerne møttes for å diskutere mulige løsninger, men de klarte ikke å bli enige om en felles strategi.

En av dem, Tom Brown, som er politisk konservativ, foreslo å bruke et nyutviklet kjemisk middel, Tri-Manganoxid. Dette stoffet var sterkt anbefalt av vitenskapelige eksperter, som hevdet at det kunne gjenopprette balansen i det globale klimaet hvis det blir sluppet ut i atmosfæren i store mengder. Matt Greene, en mer venstreorientert politiker, foreslo å tvinge gjennom strengere lover som ville hindre produksjon av skadelige produkter og begrense forbruk som ikke er bærekraftig. Bob Wayne, en politisk talsmann for frie markedskrefter, foreslo å oppheve alle begrensninger på frihandel. Han mente at markedskreftene automatisk ville resultere i den beste løsningen for alle.

Brown, Greene og Wayne gikk i gang med å finne samarbeidspartnere for å sette planene sine ut i livet. Brown klarte å få støtte fra WHO (Verdens helseorganisasjon) til bruken av Tri-Manganoxid. Greene fikk støtte fra WTO (Verdens Handelsorganisasjon) til sitt forsøk på å finne en politisk og lovfestet løsning. Wayne fant sterk støtte hos Greenpeace til å fremme full avregulering av markedet.

Sammen med sine partnere lyktes de tre politikerne i forhandlinger med politiske institusjoner om hvordan og hvor de skulle gjennomføre sine strategier for å bekjempe klimaendringene. Kinesiske eksperter ga sterk støtte til Browns tilnærming og tillot spredning av Tri-Manganoxid over store områder i Kina. Greene klarte å overbevise et flertall av europeiske politikere, noe som resulterte i at Europaparlamentet vedtok et sett lover som forbød produksjon av skadelige stoffer og sikret regulering av forbruket i EU. Wayne vant fram med sitt syn i USA, hvor han fikk etablert et fullstendig fritt marked i hele Nord-Amerika.

Men fem år senere viste det seg at alle de tre politikerne hadde fått store problemer. Det var ingen bedring i klimaproblemet, og alle strategiene hadde hatt skadelige bieffekter. Store deler av Kinas befolkning led av pusteproblemer; den europeiske økonomien var rammet av alvorlige kriser, og i Nord-Amerika økte fattigdom, diskriminering og sosiale opptøyer.

Dette tvang Brown, Greene og Wayne til å gjennomføre ytterligere tiltak i sine respektive regioner. Med tillatelse fra det kinesiske regimet innførte Brown visse frihandelstiltak i Kina. Med støtte fra EU startet Greene et program for utslipp av en viss mengde Tri-Manganoxid over Europa. Wayne ble presset av den amerikanske regjeringen til å innføre noen restriksjoner på frihandel i de nord-amerikanske markedene.

Ytterligere fem år senere kunne alle de tre politikerne vise til en viss suksess. I Kina, i Europa og i USA antydet vitenskapelige målinger at klimaendringene var i ferd med å bremse betydelig.

Det oppsto imidlertid en heftig diskusjon om hvilken strategi som var den mest effektive og som kunne tilskrives de positive resultatene. Naturlig nok krevde alle tre – Brown, Greene og Wayne – æren for den positive utviklingen, og hver av dem hevdet at det var hans originale idé som stod bak suksessen. Krangelen ble faktisk så aggressiv at til og med drapstrusler begynte å sirkulere. Greenpeace, WTO og WHO prøvde å roe ned partene.

Ifølge vitenskapelige eksperter var utslipp av Tri-Manganoxid hovedårsaken til forandringen. Økonomer ved Harvard publiserte en rapport som viste at de viktigste effektene kom av frihandel. En gruppe grasrotorganisasjoner fremskaffet bevis for de avgjørende effekter av lovfestede reguleringer.

En sommerdag i 2035 ble Brown, Greene og Wayne funnet døde på hotellrommene sine. Brown var skutt i hodet, Greene lå kvalt i sengen, og Wayne var tydelig forgiftet, med noe svart som kom ut av munnen hans.

## English Translation

In the year 2025 a trio of ambitious politicians decided to seriously tackle the problem of climate change. Disastrous storms and droughts were accumulating worldwide, leading to famines and economic depressions in large parts of the world. The three met to discuss feasible solutions, but they were not able to agree on a common strategy.

One of them, Tom Brown, a political conservative, proposed using a newly synthesized chemical compound, Tri-Manganoxid: The compound was highly recommended by scientific experts, who claimed that it could restore the global climate to normal levels when released in the atmosphere in large amounts. Matt Greene, a more politically left-wing politician, proposed enforcing stricter laws prohibiting the production of harmful products and constraining unsustainable consumption. Bob Wayne, a political advocator of the free market, proposed relaxing all restrictions on free trade. He believed that market forces would automatically yield the best solution for all.

Brown, Greene and Wayne set out to find coalition partners to implement their plans. Brown was able to find the WHO (World Health Organization) supporting the use of Tri-Manganoxid. Greene was supported by the WTO (World Trade Organization) in his efforts for a political and legal solution. Wayne found strong support from Greenpeace in forwarding a full deregulation of markets.

Together with their partners, the three politicians successfully negotiated with political institutions on how and where to implement their strategies to fight climate change. Chinese experts strongly endorsed Brown’s approach and permitted the dispersal of Tri-Manganoxid over large regions of China. Greene was able to convince a majority of European politicians, resulting in the European parliament passing a set of laws prohibiting production of harmful substances and regulating consumption in the EU. Wayne prevailed in the United States where he could establish a completely free market across North America.

However, five years later, it turned out that all three politicians had big problems. No improvements to climate change could be observed and all strategies bore harmful side effects. Large parts of the Chinese population suffered from respiratory problems; the economy suffered severe breakdowns in Europe, and North America saw an increase in poverty, discrimination, and social riots.

Out of necessity, Brown, Greene, and Wayne implemented further measures in their respective regions. Brown, with the permission of the Chinese regime, was able to introduce a certain amount of free trade in China. Greene, backed by the EU, started a program of emissions of a certain amount of Tri-Manganoxid across Europe. Wayne, pressed by the US government, introduced some restrictions on free trade in North American markets.

Another five years later, all three politicians could announce some success. In China, in Europe, and in the US, scientific measurements indicated that climate change was slowing down significantly.

However, a fierce dispute arose about which strategy was primarily effective and responsible for the positive results. Naturally, Brown, Greene, and Wayne each claimed credit for the positive development and argued that it was his original idea that eventually prevailed. In fact, the quarrel became so aggressive that even death threats were circulated. Greenpeace, the WTO, and the WHO tried to appease the opponents.

According to scientific experts, the emission of Tri-Manganoxid was the main cause of change. Economists from Harvard published a report demonstrating that major effects resulted from free trade. A group of grassroots organizations provided evidence for the pivotal effects of legal regulations.

On a summer day in June 2035, Brown, Greene, and Wayne were all found dead in their hotel rooms. Brown was shot in the head, Greene was lying in his bed strangled, and Wayne was evidently poisoned, with something black coming out of his mouth.

# Retelling Instructions

## Original Norwegian Version

### Control condition (mere recall)

*<Stage 1:* I går / *Stage 2:* For en uke siden, da du deltok i første del av denne undersøkelsen,> leste du en historie om forskjellige politiske hendelser knyttet til klimaendringer. Nå ber vi deg om å gjenfortelle historien. Skriv ned hvordan du husker historien, så fullstendig som mulig. Det gjør ingenting om du ikke er sikker på detaljene.

### Retelling to an audience

*< Stage 1:* Nedenfor finner du beskrivelsen av en person ved navn Jon. / *Stage 2:* Sist introduserte vi deg for en person ved navn Jon - i dag møter du Jon igjen.> Les beskrivelsen nøye og forsøk å se for deg så detaljert og livaktig som mulig hva slags person Jon er (for eksempel hvordan han ser ut, hvordan han tenker og føler, og hva han kan finne på å si og gjøre).

*< Insert description of Jon>*

Bruk et minutt eller to på å forestille deg hva slags person Jon er.

*< New screen>*

*< Stage 1:* I går / *Stage 2:* For en uke siden, da du deltok i første del av denne undersøkelsen,> leste du en historie om forskjellige politiske hendelser knyttet til klimaendringer. Nå ber vi deg om å gjenfortelle historien. Se for deg at du forteller historien til Jon. Skriv ned hvordan du ville fortelle historien hvis Jon hørte på. Det gjør ingenting om du ikke er sikker på detaljene.

#### Description of Jon the individualist

Jon legger stor vekt på verdier som personlig frihet og økonomisk handlefrihet. For ham er det viktig at de som arbeider hardt og har talent, får bedre betalt og har mer politisk innflytelse enn andre. Han forakter ideen om et ensartet samfunn hvor alle har det samme velstandsnivået. Han mener at menneskene har rett til å utnytte naturen, som han tror er et robust system som ikke så lett forstyrres eller ødelegges av menneskelige aktiviteter. Han mener naturen alltid vil klare å gjenfinne den best mulige balansen.

#### Description of Jon the hierarchist

Jon legger stor vekt på verdier som tradisjon, sosiale normer, hierarkier og respekt for autoritetene. For ham er det viktig at de som har betydningsfulle posisjoner i samfunnet og er anerkjente ledere og eksperter, respekteres og følges. Han forakter ideen om et ensartet samfunn hvor alle har de samme påvirkningsmulighetene, uavhengig av kompetanse og ekspertise. Han mener at mennesker kan bruke naturen til det de trenger, så lenge det gjøres på en kompetent og fornuftig måte. Han mener at naturen er ganske robust og bare kan skades og ødelegges dersom mennesker handler uten nødvendig kunnskap og innsikt.

#### Description of Jon the egalitarian

Jon legger stor vekt på verdier som likhet og sosialt likeverd. For ham er det viktig at rikdom og makt er jevnt fordelt mellom alle mennesker, og at alle tar del i politiske beslutninger. Han forakter egoistiske mennesker med stor økonomisk eller politisk makt. Han mener at menneskene har ansvar for å ta vare på naturen, ikke utnytte den, og at naturen er et nøye balansert system som lett forstyrres og ødelegges av menneskelige aktiviteter.

## English Translation

### Control condition (mere recall)

*< Stage 1:* Yesterday you read a story about various political events concerning climate change. Now we ask you to re-tell this story. *Stage 2:* A week ago, when you participated in the first part of this survey, you read a story about various political events concerning climate change. Now we ask you to re-tell this story again. > Please write down how you remember the story, as completely as possible. It does not matter if you are uncertain about details.

### Retelling to an audience

*< Stage 1:* Below you find the description of a person named Jon. Please read this description carefully / *Stage 2:* The last time we introduced a person named Jon to you - today you meet Jon once more. Please read this description carefully again > and try to imagine as detailed and vividly as possible what kind of person Jon is (for example, what he may look like, how he thinks and feels, and what he may say and do).

*< Insert description of Jon>*

Take a minute or two to imagine what kind of person Jon is.

*< New screen>*

*< Stage 1:* Yesterday you read a story about various political events concerning climate change. Now we ask you to re-tell this story. Imagine you are telling it to Jon. Please write down how you would tell the story if Jon were listening. It does not matter if you are uncertain about details. /
*Stage 2:* A week ago, when you participated in the first part of this survey, you read a story about various political events concerning climate change. Now we ask you again to re-tell this story and to imagine that you are telling it to Jon. >
Please write down how you would tell the story if Jon were listening. It does not matter if you are uncertain about details.

#### Description of Jon the individualist

Jon is a person who deeply endorses the values of personal freedom and economic liberty. For him it is important that those who work hard and are highly talented deserve to be wealthier, and should have more political influence than others. He abhors the idea of a uniform society with everybody having the same level of wealth. He thinks that humans have the right to exploit nature, which he believes is a highly robust system, not easily disrupted and destroyed by human activities. He believes that nature is always capable of returning to its optimal balance.

#### Description of Jon the hierarchist

Jon is a person who deeply endorses the values of tradition, social customs, hierarchies, and respect for authority. For him it is important that those holding important social positions, and who are acknowledged leaders as well as recognized experts, should be respected and followed. He abhors the idea of a society where everybody has the same influence, regardless of their competency and expertise. He thinks that humans are able to utilize nature for their own purposes, if done in a competent and rational way; he believes that nature is fairly robust and will only be disrupted and destroyed if people act in ways that reveal lack of necessary knowledge and insight.

#### Description of Jon the egalitarian

Jon is a person who deeply endorses the values of equality and social equity. For him it is important that wealth and power is distributed equally among all people, and that everyone is involved in political decision making. He abhors selfish individuals with great economic or political power. He thinks that humans are responsible for conserving nature and believes that they should not exploit nature, which he believes is a system in very delicate balance, easily disrupted and destroyed by human activities.

# World View Measure

*The items of the world view measure were adapted from Grendstad (2001), Table 1:*

*Grendstad, G. (2001). Nordic cultural baselines: Accounting for domestic convergence and foreign policy divergence. Journal of Comparative Policy Analysis: Research and Practice, 3(1), 5-29. https://doi.org/10.1080/13876980108412652*

*Order of the items was randomized for each participant.*

## Original Norwegian Questionnaire

*Response scale for each item (radio buttons):*

| *Svært uenig* | *Uenig* | *Noe uenig* | *Verken uenig eller enig* | *Noe enig* | *Enig* | *Svært enig* |
| --- | --- | --- | --- | --- | --- | --- |

Nå vil vi gjerne spørre deg om hvordan du ser på livet og samfunnet generelt. Vennligst les påstandene nedenfor. Angi hvor enig eller uenig du er i hver påstand.

- Jeg føler at livet er et lotteri. *[Fatalism]*
- Dersom noen har visjoner og evner til å skaffe seg materielle goder, burde de få lov til å glede seg over dem. *[Individualism]*
- Vi må redusere ulikhetene mellom kvinner og menn. *[Egalitarianism]*
- Markedskonkurranse er nesten alltid den beste måten å dekke folks behov på. *[Individualism]*
- I et rettferdig system bør de flinkeste tjene mest penger. *[Individualism]*
- Vi tar best vare på de kommende generasjoner ved å holde fast på våre tradisjoner og skikker. *[Hierarchism]*
- Uansett hvilket parti man stemmer på, så fortsetter alt som før. *[Fatalism]*
- Samfunnet fungerer best når folk følger alle lover og regler nøye. *[Hierarchism]*
- Framtiden er for usikker til at det er mulig å legge reelle planer. *[Fatalism]*
- Alle bør ha like muligheter til å lykkes eller feile uten at myndighetene blander seg inn. *[Individualism]*
- De fleste mennesker skaffer seg venner bare fordi de har nytte av dem. *[Fatalism]*
- Beslutninger i politikken bør i større grad baseres på folkelig deltakelse. *[Egalitarianism]*
- Et av problemene med folk i dag er at de altfor ofte setter seg opp mot autoritetene. *[Hierarchism]*
- Folk som har suksess i næringslivet har rett til å bruke rikdommen sin slik de selv har lyst til. *[Individualism]*
- Det ville bli fredeligere i verden dersom rikdommen var jevnere fordelt mellom landene. *[Egalitarianism]*
- Vårt samfunn trenger en større grad av rettferdighet slik at rikdommen kan fordeles jevnere. *[Egalitarianism]*
- Samarbeid med andre mennesker fungerer sjelden. *[Fatalism]*
- Folk lever mer harmonisk sammen når forskjellige typer mennesker har hver sin plass i samfunnet. *[Hierarchism]*
- Jeg er tilhenger av en skattereform som legger en større belastning på bedrifter og personer med høye inntekter. *[Egalitarianism]*
- Respekt for autoriteter er en av de viktigste tingene barn bør lære seg. *[Hierarchism]*

## English Translation of Questionnaire

*Response scale for each item (radio buttons):*

| *Disagree strongly* | *Disagree* | *Disagree somewhat* | *Neither agree nor disagree* | *Agree somewhat* | *Agree* | *Agree strongly* |
| --- | --- | --- | --- | --- | --- | --- |

Now we would like to ask you about your views on life and society in general. Please read the following statements. Please indicate for each statement how much you agree or disagree with it.

- I feel that life is a lottery. *[Fatalism]*
- If people have the vision and ability to acquire property, they ought to be allowed to enjoy it. *[Individualism]*
- We need to reduce inequalities between men and women. *[Egalitarianism]*
- Competitive markets are almost always the best way to supply people with things they need. *[Individualism]*
- In a fair system, people with more ability should earn more. *[Individualism]*
- The best way to provide for future generations is to preserve the customs and practices of our past. *[Hierarchism]*
- It seems that whichever party you vote for, things go on pretty much the same. *[Fatalism]*
- Society works best when people obey all rules and regulations. *[Hierarchism]*
- The future is too uncertain for a person to make serious plans. *[Fatalism]*
- Everyone should have an equal chance to succeed and fail without government interference. *[Individualism]*
- Most people make friends only because friends are useful for them. *[Fatalism]*
- Decisions in politics should rely more heavily on popular participation. *[Egalitarianism]*
- One of the problems with people today is that they challenge authority too often. *[Hierarchism]*
- People who are successful in business have a right to enjoy their wealth as they see fit. *[Individualism]*
- The world would be a more peaceful place if its wealth were divided more equally among nations. *[Egalitarianism]*
- Our society needs a larger degree of fairness to make the distribution of goods more equal. *[Egalitarianism]*
- Cooperation with others rarely works. *[Fatalism]*
- People live together more harmoniously when different types of people have their own place in society. *[Hierarchism]*
- I support a tax shift so that burden falls more heavily on corporations and people with large incomes. *[Egalitarianism]*
- Respect for authority is one of the most important things that children should learn. *[Hierarchism]*
